# Blood Mucorales PCR to track down *Aspergillus* and Mucorales co-infections in at-risk hematology patients: A case-control study

**DOI:** 10.3389/fcimb.2022.1080921

**Published:** 2022-12-08

**Authors:** Robina Aerts, Sien Bevers, Kurt Beuselinck, Alexander Schauwvlieghe, Katrien Lagrou, Johan Maertens

**Affiliations:** ^1^ Department of Internal Medicine, University Hospitals Leuven, Leuven, Belgium; ^2^ Department of Microbiology, Immunology and Transplantation, KU Leuven, Leuven, Belgium; ^3^ Department of Haematology, University Hospitals Leuven, Leuven, Belgium; ^4^ Department of Laboratory Medicine, University Hospitals Leuven, Leuven, Belgium; ^5^ Department of Haematology, AZ Sint-Jan Brugge, Bruges, Belgium

**Keywords:** Mucorales PCR, invasive mould infections, aspergillosis, mucormycosis, mould co-infections, difficult to cure infections, Mucorales, *Rhizopus*

## Abstract

**Introduction:**

Serum Mucorales PCR can precede the final diagnosis of invasive mucormycosis by several days or weeks and could therefore be useful as a non-invasive screening tool.

**Methods:**

We assessed the performance of a commercial Mucorales PCR assay (MucorGenius®, PathoNostics, Maastricht, The Netherlands) on prospectively collected banked sera from hematology patients at risk for invasive mould infections. We evaluated if there is an underestimated incidence of missed Mucorales co-infections in patients with invasive aspergillosis (IA). We tested Mucorales PCR on the sera of all patients with a diagnosis of at least possible IA (EORTC-MSGERC consensus criteria) before the start of any antifungal therapy, and in a control group of similar high-risk hematology patients without IA (in a 1:4 ratio). When a positive Mucorales PCR was observed, at least 5 serum samples taken before and after the positive one were selected.

**Results:**

Mucorales PCR was performed in 46 diagnostic serum samples of cases and in 184 controls. Serum Mucorales PCR was positive in 4 cases of IA (8.7%; 12.9% of probable cases) and in 1 control case (0.5%) (p=0.0061, OR=17.43 (1.90-159.96). Post-mortem cultures of the positive control became positive for Rhizopus arrhizus. Mortality of IA cases with and without a positive Mucorales PCR was not significantly different. Only in the PCR positive control case, serial serum samples before and after the diagnostic sample were also positive.

**Discussion:**

It is not entirely clear what a positive Mucorales PCR in these cases implies since the 4 Mucorales PCR positive cases were treated with antifungals with activity against Mucorales. In addition, PCR was positive only once. This study does not provide enough evidence to implement Mucorales PCR screening. However, our findings emphasize once more the importance of considering the possibility of dual mould infections, even in patients with a positive galactomannan detection.

## Introduction

Patients with prolonged, profound neutropenia and allogeneic hematopoietic cell transplant (HCT) recipients are at increased risk of invasive mould diseases (IMD), including invasive aspergillosis (IA) and invasive mucormycosis (IM). In the absence of a microbiological finding (e.g., a positive culture), these two fungal entities are often undistinguishable. Despite the recent availability of new broad-spectrum antifungal drugs and substantial improvements in diagnosing IMD over the last two decades, their management remains complex. At present, the all-cause mortality rate of IM ranges between 40% to 80%, largely depending on underlying conditions and sites of infection. The poorest outcome is observed in patients with hematological malignancies and HCT recipients (Cornely et al., 2019). Disseminated disease, especially involving the central nervous system (CNS), is often associated with mortality rates exceeding 80%. In addition, co-infection with other fungi also increases mortality (Marty et al., 2018). Suspected mucormycosis requires urgent intervention, especially given the rapidly progressive and destructive nature of the infection (Katragkou et al., 2014). Delayed initiation of appropriate therapy is associated with increased mortality.

As with other fungal diseases, the overall survival can be significantly improved by an earlier diagnosis. In addition, and specifically for IM, a multidisciplinary approach involving (in many cases) surgical debridement, control of the underlying disease and the use of polyene antifungals is of paramount importance. However, current diagnostic tools, such as culture, lack sensitivity (Skiada et al., 2013; Cornely et al., 2019). Hence, mucormycosis is often not diagnosed premortem or suspected (too) late during the course of the disease, e.g. when a patient with suspected invasive aspergillosis is not improving while receiving voriconazole therapy (Bergantim et al., 2013).

Recently, several PCR assays for the detection of Mucorales DNA have been developed (Millon et al., 2016; Mercier et al., 2019; Guegan et al., 2020; Skiada et al., 2020). Mucorales PCR detection in plasma or serum samples could be an interesting non-invasive test for screening purposes to trigger early intervention (Guegan et al., 2020).

This study aims to evaluate the performance of a commercially available Mucorales PCR assay (MucorGenius^®^, PathoNostics, Maastricht, The Netherlands) as a screening tool on prospectively collected and stored sera from a cohort of at-risk haematology patients. If detection of circulating Mucorales DNA proves to be a good and fast diagnostic test that precedes the final clinical diagnosis, this test could be especially useful as a non-invasive screening tool in patients with unresponsive or relapsing fever or in cases in which sampling for culture or histopathology is not feasible. With an IA co-infection rate of up to 25%, we want to evaluate if there is an underestimated incidence of Mucorales infections in patients with aspergillosis (Millon et al., 2016; Scherer et al., 2018; Guegan et al., 2020).

Therefore, the objectives of this study were to assess the performance of Mucorales-specific PCR testing in high-risk hematology patients, to assess the incidence of IM in these patients, specifically the rate of missed diagnosis, and the difference of IM incidence in patients with IA versus in patients without IA. As a second objective we wanted to compare the survival difference in patients with IA with and without positive Mucorales PCR in the diagnostic sample. Third, in patients with positive Mucorales PCR, we wanted to investigate the kinetics of the Mucorales PCR with or without adequate treatment.

Since early diagnosis of IM has been shown to improve survival, an early diagnostic test may potentially result in a survival benefit. In addition, early confirmation may reduce the number of unnecessary diagnostic test and antimicrobial drugs, thereby reducing hospital cost. Finally, a better understanding of the incidence of Aspergillus and Mucorales co-infections will lead to a better approach towards a patient with an invasive mould disease, with a smaller delay to adequate treatment.

## Materials and methods

The aim of our study was primarily to assess the incidence of IM as well as the rate of missed cases – or delayed diagnoses – in a large cohort of at-risk hematology patients. In addition, we wanted to assess the incidence of co-infections between IA and IM, and to assess if underlying mucormycosis could explain a possible deterioration under the initial treatment for aspergillosis.

### Data and sample collection

The sample collection was performed between January 2017 and April 2021 at the Belgian National Reference Centre for Mycosis (University Hospitals Leuven, Leuven, Belgium). We prospectively screened consecutive adult patients (≥18 years of age) with an underlying haematological disorder at risk for developing IA. The population included patients receiving (1) intensive chemotherapy for acute leukemia or high-risk myelodysplastic syndromes, (2) hematopoietic cell transplant recipients (up to 1 year after transplantation), and (3) patients with severe aplastic anemia receiving anti-thymocyte globulin (ATG)–based immunotherapy.

All these patients received fluconazole (400 mg/day) with daily screening for serum galactomannan (GM) detection, using the Platelia assay (Bio-Rad, Marnes-la-Coquette, France). Anti-mould drugs were not given, prophylactically nor empirically. After 98–120 hours of fever unresponsive to broad-spectrum antibiotics or clinical signs or symptoms suggestive of pulmonary disease (e.g., dry cough, pleuritic chest pain, or hemoptysis) or serum GM detection (2 consecutive GM indices ≥0.5 or 1 GM index ≥0.8), a computed tomography (CT) scan of the chest was performed. In case of any radiological abnormality, bronchoscopy with bronchoalveolar lavage (BAL) was performed for extensive microbiological testing (including GM detection using an index threshold of ≥1.0) and microscopic analysis.

After inclusion, we prospectively collected and banked serum samples twice weekly whenever the patient was hospitalized or during outpatient visits. Once antifungal therapy was initiated for IA, serum sampling continued twice weekly up to 6 weeks after diagnosis.

Patients were excluded from the study if they were receiving treatment for a previous fungal disease or if they had completed antifungal therapy less than 6 weeks before the time of enrollment. This biobanking project was approved by the Ethics Committee Research UZ/KU Leuven (S59863/S61797, NCT03004092). All subjects provided written informed consent before any study-specific procedure.

### Study design and participants

We retrospectively tested Mucorales PCR on the stored sera of all patients with a diagnosis of at least possible IA [based on radiological criteria following the 2020 EORTC/MSGERC consensus criteria (Donnelly et al., 2020)] before the start of any antifungal therapy (‘the cases’), and in a control group of similar high-risk hematology patients without IMD (‘the controls’). Patients who were suspected of having IA because of fever or because they underwent a diagnostic workup, but never received anti-Aspergillus therapy due to lack of supportive evidence were considered as controls.

For cases, the serum sample taken at diagnosis before the start of anti-*Aspergillus* therapy was used for Mucorales PCR. We selected all cases of IA for whom at least 1000 µL of diagnostic serum sample was available (17 patients with IA were excluded). For controls the serum sample that was drawn on the day that the patient had one of the following clinical events (in descending order of likelihood for IFD): on the day of bronchoscopy, day of high-resolution CT scanning of the chest, or on the day that the patient developed a fever of greater than 38.2°C. Patients not having one of these events were excluded from further analysis. For each case of IA, we selected 4 high-risk controls of whom at least 1000 µL of serum on the day around the clinical event was available.

When a positive Mucorales PCR was observed, at least five serum samples taken before and five serum samples taken after the positive sample in time were selected, to assess the kinetics (and the relation with outcome) and to have a better idea of the diagnostic delay when relying only on conventional tools. In case the earliest and/or latest samples in this time window were positive by PCR, we extended our search period until the samples became negative, or until there were no more stored samples available in the biobank.

For each patient, we collected age, date of diagnostic sample, antifungal therapy, underlying disease, site of infection and time and cause of death, as well as the date of the first EORTC/MSGERC defined clinical signs (i.e., radiological features or endoscopic findings). This study, as part of the biobanking project, was separately approved by the Ethics Committee Research of University Hospitals Leuven (S65530-July 2, 2021).

### Definitions

Patients were classified by two independent physicians as having proven IA, probable IA or possible IAas per recently updated European Organization for Research and Treatment of Cancer (EORTC) and the Mycoses Study Group Education and Research Consortium (MSGERC) consensus definitions (Donnelly et al., 2020). We added a classification of “suspected” IA for patients with a mycological criterion, but with imaging features other than nodule, halo sign or air crescent sign. For the classification of patients, BAL GM was considered to be positive when (a) the optical density index (ODI) was ≥ 0.8, if the concomitant serum GM was positive (ODI ≥ 0.7), or (b) an ODI ≥ 1.0 if the serum GM was negative or unavailable

Adequate therapy against Mucorales infections was defined as liposomal amphotericin B ≥ 5 mg/kg, posaconazole or isavuconazole.

### DNA extraction and qPCR assay

DNA was extracted from 1000 µL of the serum sample using the NucliSens easyMAG/eMAG (BioMérieux, France) according to the manufacturer’s instructions. Briefly, samples were pretreated and lysed in a GuSCN-buffer, followed by binding of the free nucleic acid to silica coated magnetic beads. The beads were then washed, after which the bound nucleic acid was eluted into a final volume of 50 µL. The MucorGenius^®^ assay (PN-700, PathoNostics, Maastricht, The Netherlands) was then run using 5 µL of the extracted DNA on a LightCycler 480II (Roche, Basel, Switzerland). This assay contains a premix of Mucorales specific primers for real-time PCR as well as an internal control for quality assurance. The PCR detects DNA of *Rhizopus* species, *Mucor* species, *Lichtheimia* species, *Cunninghamella* species and *Rhizomucor* species. Sensitivity of the test in previous studies was 75% (89% in hematology patients and 86% when only considering proven mucormycosis) in peripheral blood and 90% in pulmonary specimens. Specificity was 97.9%-100% (Mercier et al., 2019; Guegan et al., 2020).

The cycle threshold (Cq) was determined using the fit point method. Any Cq > 40 was set to a fixed Cq of 40. Any detectable amount of DNA (i.e., Cq < 40) was considered a positive result. Positive and negative controls were included in every batch. The turnaround time of the test including DNA extraction was around 3.5 h.

### Statistical analysis

Fisher’s exact test was used to compare the rate of positivity between the cases of IA (cases) and high-risk hematology patients without IA (control) group. Calculated p-values are two-sided, and p-values less than 0.05 are considered statistically significant. Survival curves were made by Kaplan-Meier method, compared using the log-rank test. Adjusted survival analysis was done with Cox proportional hazard regression. Analyses were performed with the statistical software R (Version 1.3.1093).

## Results

### Patient population

**Figure 1:**
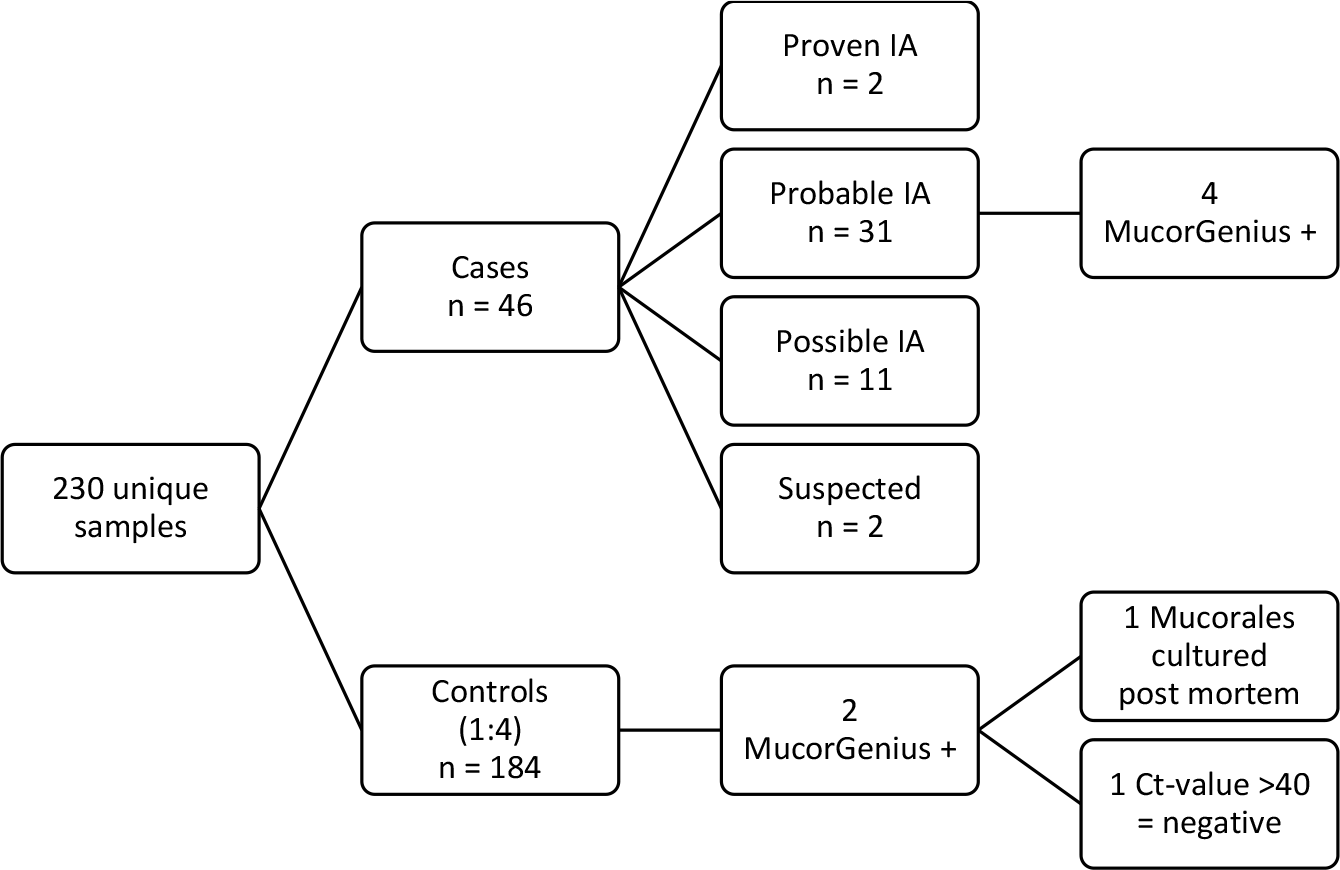
Overview of samples tested with Mucorales PCR. Cases were defined based the 2020 EORTC/MSGERC consensus criteria (Donnelly et al., 2020). Controls were patients that underwent a diagnostic workup but never received anti-Aspergillus therapy due to lack of supportive evidence. For cases, the serum sample taken at diagnosis before the start of anti-Aspergillus therapy was used, for controls the serum sample that was drawn on the day that the patient had a clinical event (bronchoscopy or chest CT).

Mucorales PCR was retrospectively performed on banked sera from 230 haematology patients: in 46 diagnostic sera of cases and in 184 (1:4) sera taken at the time of a diagnostic event in controls. In total Mucorales PCR was performed on 285 serum samples. Characteristics of the study cohort are summarized in **Table 1**. In this initial analysis, Mucorales PCR was positive in 4 cases of invasive aspergillosis (8.7%). According to the EORTC/MSGERC criteria, 2/46 patients had proven IA, 31/46 probable IA and 11/46 possible IA. Two cases were classified as suspected IA. Mucorales PCR was positive in 4/31 (12.9%) cases of probable IA. Mucorales DNA was detected in 2 controls in whom no IA was diagnosed, Mucorales PCR was positive in 1 of them (0.5%), in the other control Cq was >40.0 and was therefore concluded to be negative (**Figure 1**).

**Table 1:** Characteristics of the hematology patients included in the study cohort.

	Cases (%)(n = 46)	Controls (%)(n = 184)	p-value
Age (median, range)	62 (range 20-83)	58 (range 21-77)	0.43
Gender			0.17
Male	26 (56.5)	125 (67.9)	
Female	20 (43.5)	59 (32.1)	
Neutropenia	35 (76.1)	40 (21.7)	**<0.0001**
Use of steroids	18 (39.1)	15 (8.2)	**<0.0001**
Previous allogeneic HCT	15 (32.6)	27 (14.7)	**0.0093**
GvHD	6 (13.0)	8 (4.3)	**0.039**
T-lymphocyte inhibitor	12 (26.1)	69 (37.5)	0.17
Hemodialysis	1 (2.2)	3 (1.6)	1
Diabetes	1 (2.2)	12 (6.5)	0.47

HCT, hematopoietic cell transplantation; GvHD, graft-versus-host disease.

p-values <0.05 in bold (denote statistical significance).

Median Cq value of the PCR positive samples was 32.5 cycles (range 29.4-36.7). The occurrence of a positive Mucorales PCR in the event sample of these high-risk hematology patients – irrespective of the clinical relevance – was significantly different between patients with IA and patients without IA (p=0.0061, OR=17.43 (1.90-159.96)).

### Characteristics of IA cases

**Figure 2:**
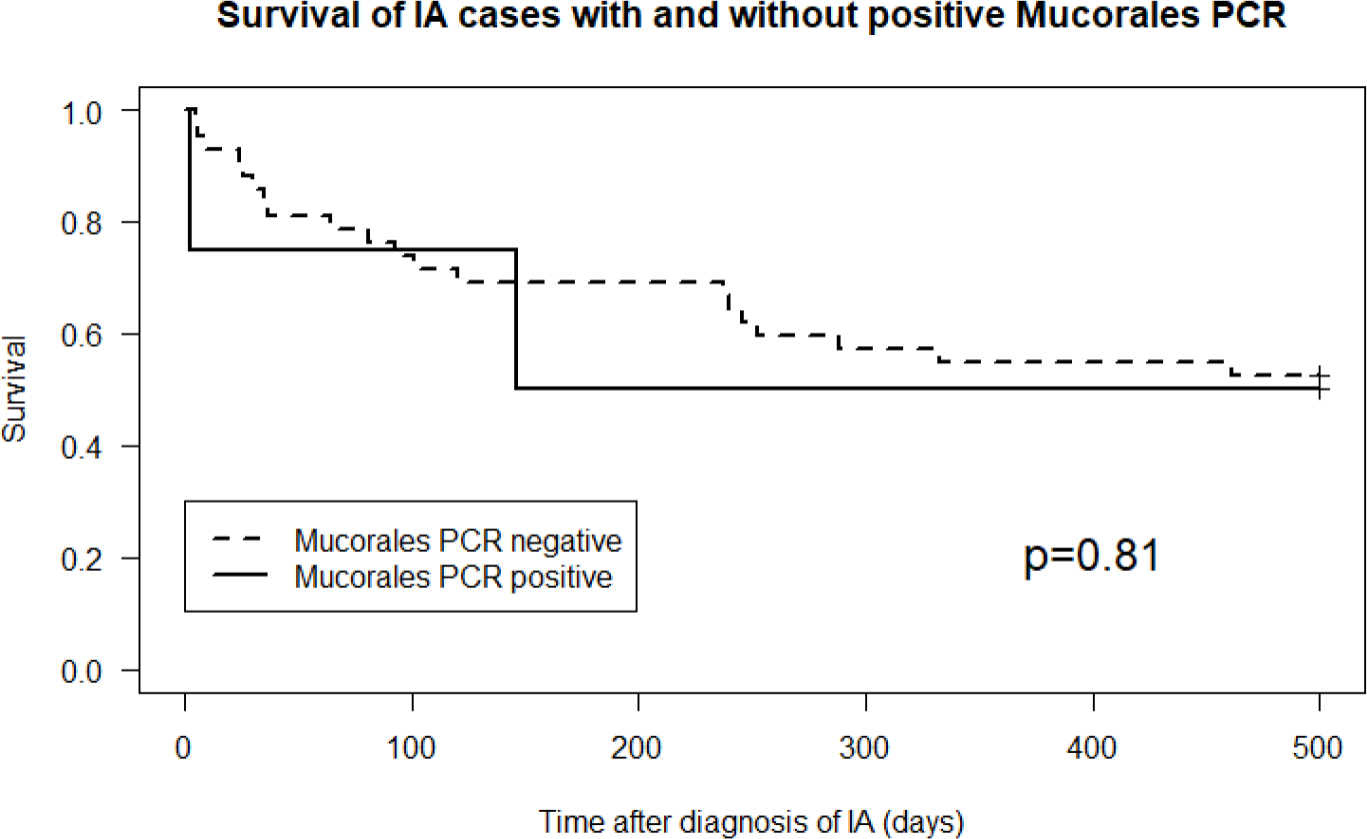
Cumulative survival in cases of invasive aspergillosis with versus without a positive Mucorales PCR (p=0.81).

Characteristics of all the cases of IA and those who had a positive Mucorales PCR in the diagnostic serum sample are summarized in **Supplementary Tables 1** and **2**. The one patient in the control group that had a positive Mucorales PCR, died soon thereafter. Retrospectively, cultures became positive for Mucorales post-mortem (bronchoalveolar lavage fluid (BALf): Mucorales species; bronchus aspirate: *Rhizopus arrhizus*), defining a probable IM. Mortality of cases IA with and without a positive Mucorales PCR was not significantly different (p=0.81, **Figure 2**).

### Kinetics

**Figure 3:**
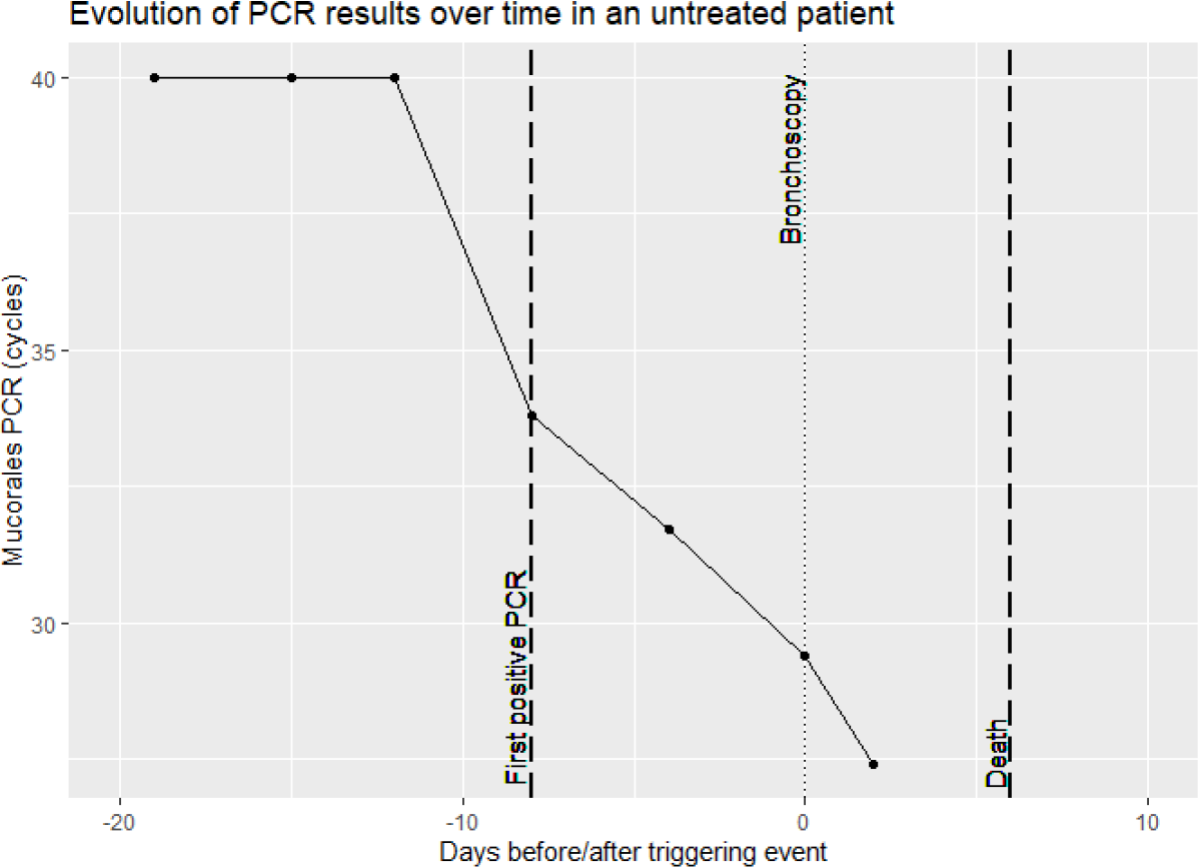
Overview of Mucorales PCR results in serial serum samples of a patient not receiving anti-mold treatment.

**Figure 4:**
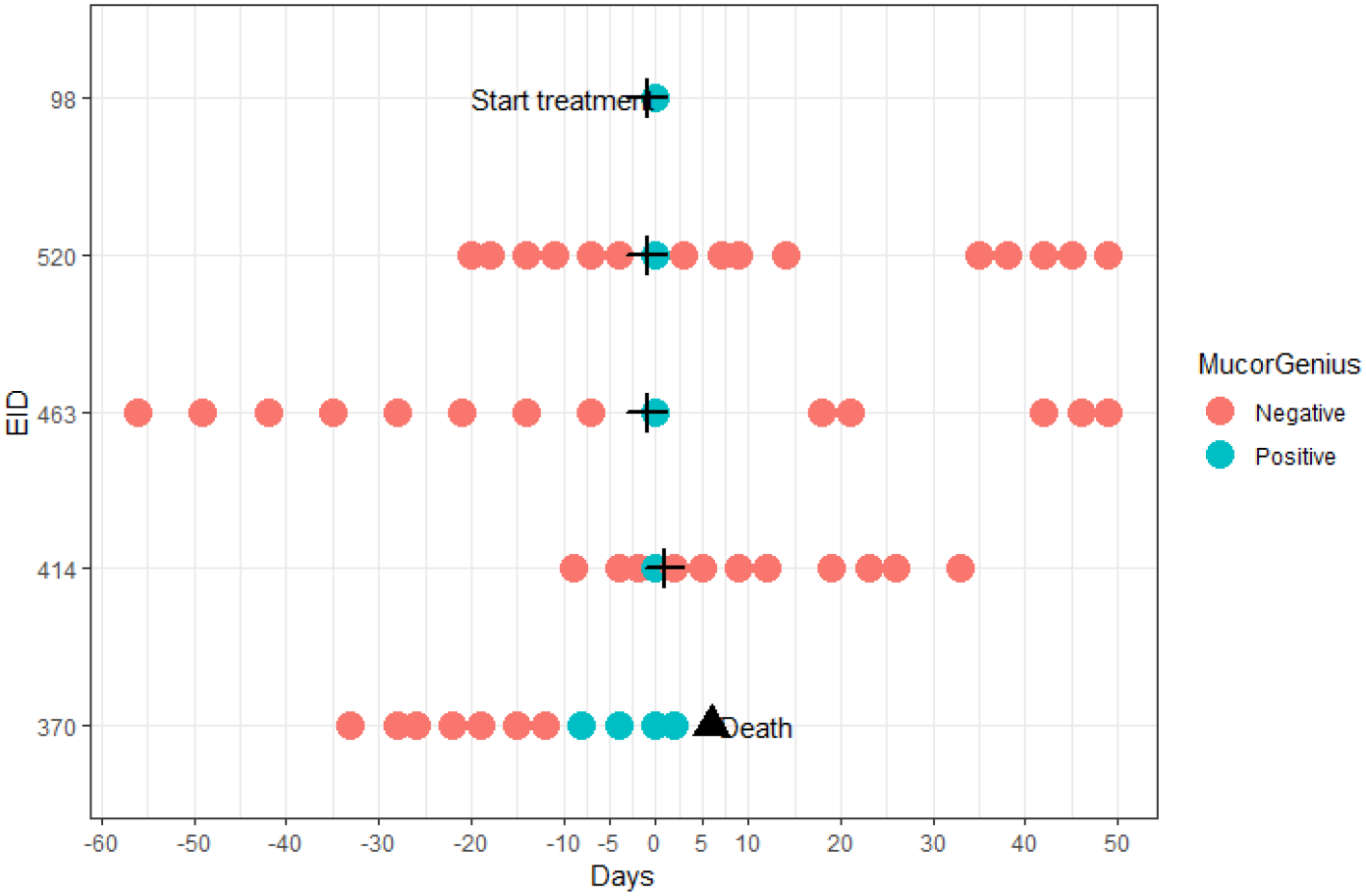
Temporal overview of the additional tested samples of patients with a positive Mucorales PCR in the diagnostic sample. The cross indicates start of anti-mold therapy; the triangle denotes death of a patient.

Only in 1 Mucorales PCR positive patient (the one from the control group), serum samples in time before and after the diagnostic sample were also positive. This patient did not receive antimould therapy. Evolution of the PCR in this patient is visualized in **Figure 3**. For 1 case no serial samples were available. In the other 3 cases, there was only 1 unique positive serum sample. Treatment for IA in these 3 patients was started around the diagnostic sample. Treatment consisted of amphotericin B, isavuconazole or posaconazole, suggesting the possibility of immediate clearance of Mucorales PCR under treatment (**Figure 4**), or at least a situation in which cannot be differentiated between a false positive, spontaneous clearance or adequate treatment.

## Discussion

Predisposing factors for both pulmonary mucormycosis and IA in haematology patients are similar and co-infections have been reported in 20-25% of mucormycosis cases (Millon et al., 2016; Scherer et al., 2018; Skiada et al., 2020). It is therefore not surprising that a positive Mucorales PCR is found more frequently in cases of IA than in the control group of high-risk hematology patients. Our results were in line with findings by Guegan et al. where six of 63 patients (9.5%) diagnosed with probable IA (alone), according to EORTC/MSGERC criteria, had at least one positive mucorales PCR when performed on pulmonary samples (BAL, tracheal aspiration, sputum, pleural fluid and lung biopsy) (Guegan et al., 2020).

As per current EORTC/MSGERC definitions (Donnelly et al., 2020), the cases of IA with positive Mucurales PCR would not be defined as cases of IM because culture and/or biopsy was negative. If Mucorales PCR would have been an accepted mycological criterium for diagnosis of probable IM, the four patients would have had the disease. However, its clinical significance remains unclear since mortality was not different between the cases of IA with and those without positive Mucorales PCR. However, these four patients were treated with either Amphotericin B, isavuconazole or posaconazole, drugs with well-known activity against Mucorales (Cornely et al., 2019). In addition, PCR was only positive once. Do we have to see this as an unsignificant ‘bleb’, or does this mean detectable Mucorales DNA which is cleared immediately after start of adequate treatment?

In the retrospective study by Millon et al. time to negativity was 7 (3-19) days (Millon et al., 2016), but this timing seemed to depend more on the availability of samples (no daily sampling available for all of the cases). Moreover, in the cases in which PCR became negative, the Ct-value of the second sample after the start of therapy was usually close the 40 cycles. Cq-values in the study of Millon et al. are similar our results.

Guegan et al. used serum PCR in patients with a positive PCR on pulmonary samples to consider the samples as truly positive (Guegan et al., 2020). PCR was not performed on pulmonary samples of our patients, but in a study by Scherer et al. out of 24 patients with Mucorales PCR positive on BALf, 17 patients had a positive serum PCR and in 15/17 serum was the earliest to become positive (Scherer et al., 2018). An important difference between our results and the results of Guegan et al. is that in their cohort survival between PCR positive and PCR negative patients was significantly different.

False positive PCRs are rare. False positive Aspergillus PCR has been described in up to 5.9% of serum samples, coming from contamination of the sample during processing or from translocation of fungal components from the gut (White et al., 2015; Millon et al., 2019; Egger et al., 2020). The results of the Mucorales PCR in general exceed the Aspergillus PCR results because a higher number of repeats targeted, presence of multiple nuclei providing more fungal DNA and more vascular invasion in IM (Millon et al., 2016). Given the risk profile of our patients, clinicians might consider these positive Mucorales PCR results as indicative of IM missed by conventional cultures, or they might consider this a trigger for starting with an antifungal drug that also covers Mucorales species.

While the EORTC/MSGERC microbiological criteria do not include the results of Mucorales PCR, Aspergillus PCR is included, but it has its limitations. It is proposed to be combined with other antigen-based biomarkers such as GM (Egger et al., 2020). This would not be possible for Mucorales infections. Also, combining positive PCR on BALf would not help to increase specificity.

Chest CT from 3 of the 4 Mucorales PCR positive cases showed more than 10 noduli, which has previously been suggested to be more frequent in IM (Skiada et al., 2013). Other radiological findings that could be more frequent in IM are the reversed halo sign, present in none of the 4 patients [this often presents early in the disease and would probably have already triggered the clinician to cover Mucorales (White et al., 2015)], and pleural effusion, present in 2 out of 4 patients. In addition, previous prophylaxis with voriconazole could prone to IM, however, none of the patients in our cohort received anti-mould prophylaxis or anti-mould treatment ≥2 days before sampling. Also, sinus involvement is more frequent, which was the case in none of the PCR positive patients, but we know in haematology patients lung involvement remains the most frequent presentation. Time between bronchoscopy and chest CT is around 1-2 days in our cohort. Pathology was not performed on these patients, except from cytology on BALf of 1 patient which was normal.

This study probably does not provide enough evidence to implement Mucorales PCR screening. However, with a significant difference of Mucorales infections in cases of IA versus controls (an odds ratio of 17), our findings emphasize once more the importance of considering the possibility of dual mould infections, even in patients with a positive serum or BAL galactomannan detection (Cornely et al., 2019).

This study has its limitations. Other mechanisms that increase the risk of Mucorales infections in patients with IA, apart from the same underlying risk factors, have been described. Breakthrough infections occur under treatment with voriconazole and echinocandins probably because of prolonged treatment, increased survival in these patients, and because voriconazole could increase the virulence of Mucorales (Skiada et al., 2020). Because we only analysed Mucorales PCR at the for IA diagnostic sample, late co-infections could be missed. Breakthrough infections under posaconazole are also possible (Auberger et al., 2012). Analysing samples in a later stage of IA could be interesting, however this timing probably falls outside the scope of an interesting screening window.

To use Mucorales PCR as a screening tool, a high sensitivity and negative predictive value is necessary. This study does not provide sufficient information to assess this. Because our analysis starts from patients diagnosed with IA, we cannot conclude that treatment for IM could have been started earlier, or a treatment switch would be beneficial after the use of Mucorales PCR. Since results from a comparative study on Aspergillus PCR suggest that sensitivity may be higher in plasma than in serum, it is possible that our study underestimated the performance of the Mucorales qPCR, but testing in serum could be more specific (White et al., 2015; Mercier et al., 2019). Finally, the retrospective nature of the study. Performing a prospective project in which patients are monitored with daily or biweekly PCR would provide more information.

## Conclusion

Mucorales PCR could be implemented in combination with Aspergillus screening within the global management of targeted patients with a high risk of invasive mold infections. If a patient with IA improves under initial therapy together with a single negative blood Mucorales PCR test result, changing to or adding therapy covering Mucorales can be safely withheld, resulting in a reduction of unnecessary use of antifungal agents and therefore a lower number of patients exposed to potential drug toxicity. However, more information is necessary to decide on the value of this approach.

## Data availability statement

The original contributions presented in the study are included in the article/**Supplementary Material**. Further inquiries can be directed to the corresponding author.

## Ethics statement

This study involving human participants is reviewed and approved by Ethics Committee Research UZ/KU Leuven: S61979 / S65530. The patients/participants provided their written informed consent to participate in this study.

## Author contributions

Each author has made substantial contributions to the conception or acquisition of the study or to the analysis or interpretation of the data and substantively revised the work. We describe contributions to the paper using the CRediT taxonomy (Brand et al., 2015). Writing and Original Draft: RA and JM; Review and Editing: AS and KL; Conceptualization: JM; Investigation: RA, SB, and KB; Methodology: RA, KL, and JM; Formal Analysis: RA and JM. All authors contributed to the article and approved the submitted version.

## Funding

Financial support to perform the PCR testing was provided by Pfizer Inc. However, no conflicts of interest are in place. There is no involvement of the company during the sample collection, measurements and the analysis itself. Also, the necessary test material is not coming from the supporting company and they have no financial benefit in completing the study successfully.

## Conflict of interest

KL received consultancy fees from Pfizer, Abbott, MSD; and SMB Laboratoires Brussels, received travel support from Pfizer and MSD; and received speaker fees from Gilead, MSD, Roche, Abbott. JM received research grants from Merck/MSD, Gilead Sciences and Pfizer; is a consultant to Astellas, Basilea, Bio-Rad, Merck/MSD, Pfizer, Schering-Plough, F2G, Gilead Sciences, Cidara, Scynexis, Amplyx, and Luminex; and served on the speaker’s bureau of Astellas, Gilead Sciences, Bio-Rad, Merck/MSD, Pfizer, Schering-Plough, Basilea, and Viropharma/Shire.

The remaining authors declare that the research was conducted in the absence of any commercial or financial relationships that could be construed as a potential conflict of interest.

## Publisher’s note

All claims expressed in this article are solely those of the authors and do not necessarily represent those of their affiliated organizations, or those of the publisher, the editors and the reviewers. Any product that may be evaluated in this article, or claim that may be made by its manufacturer, is not guaranteed or endorsed by the publisher.

## Supplementary material

Supplementary Table 1 Description of all the cases of invasive aspergillosis tested with Mucorales PCR. IA = invasive aspergillosis; GM = galactomannan; ODI = optical density index; BDG = beta-D-glucan; AsperGenius = Aspergillus PCR (AsperGenius^®^, PathoNostics, Maastricht, The Netherlands); EORTC response at week 6 as for Segal et al. (2008); ALL = acute lymphocytic leukemia; AML = acute myeloid leukemia; MDS = myelodysplastic syndrome; CML = chronic myeloid leukemia; NP = not performed.

Supplementary Table 2 Additional information regarding the patients (4 cases of invasive aspergillosis, 1 control patient) who had a positive Mucorales PCR in the diagnostic serum sample. BDG = beta-D-glucan; serumGM = galactomannan in serum; GalactoBAL = galactomannan in bronchoalveolar lavage fluid, AsperGenius = Aspergillus PCR (AsperGenius^®^, PathoNostics, Maastricht, The Netherlands); NP = not performed.
