# Supplementary material for: Blood Mucorales PCR to track down Aspergillus and Mucorales co-infections in at-risk hematology patients: A case-control study
Source: Front Cell Infect Microbiol. 2022 Dec 8;12:1080921. doi: 10.3389/fcimb.2022.1080921 (PMC9774025; doi:10.3389/fcimb.2022.1080921)
Supplement: Supplementary Table 1 — Description of all the cases of invasive aspergillosis tested with Mucorales PCR. IA = invasive aspergillosis; GM = galactomannan; ODI = optical density index; BDG = beta-D-glucan; AsperGenius = Aspergillus PCR (AsperGenius®, PathoNostics, Maastricht, The Netherlands); EORTC response at week 6 as for Segal et al. (2008); ALL = acute lymphocytic leukemia; AML = acute myeloid leukemia; MDS = myelodysplastic syndrome; CML = chronic myeloid leukemia; NP = not performed. [file Table_1.docx]

Supplementary table 1

*Description of all the cases of invasive aspergillosis tested with Mucorales PCR. IA = invasive aspergillosis; GM = galactomannan; ODI = optical density index; BDG = beta-D-glucan; AsperGenius = Aspergillus PCR (AsperGenius®, PathoNostics, Maastricht, The Netherlands); EORTC response at week 6 as for Segal et al.* [17]*; ALL = acute lymphocytic leukemia; AML =* *acute myeloid leukemia; MDS = myelodysplastic syndrome; CML = chronic myeloid leukemia; NP = not performed.*

| **Case of IA** | **Age**  **(Years)** | **Sex** | **Underlying  Disease** | **GM**  **serum**  **(ODI)** | **GM**  **BALf**  **(ODI)** | **BDG**  **(pg/mL)** | **AsperGenius** | **Culture**  **BALf** | **Localization** | **Classification** | **EORTC response at week 6** | **Antimould Therapy** | **Mucorales PCR**  **(MucorGenius)** |
| --- | --- | --- | --- | --- | --- | --- | --- | --- | --- | --- | --- | --- | --- |
| 1 | 46 | M | ALL | 3,5 | 0,1 | NP | NP | Negative | Lungs | Suspected | Death | Amphotericin B | - |
| 2 | 78 | M | ALL | 5,2 | 4,2 | 500,00 | NP | *Aspergillus fumigatus complex* | Lungs | Probable IA | Death | Amphotericin B | **+** |
| 3 | 65 | M | AML | 0,1 | 0,1 | <31,00 | NP | Negative | Lungs | Possible IA | Partial | Caspofungine | - |
| 4 | 55 | M | AML | 1,1 | NP | <31,00 | NP | NP | Lungs | Probable IA | Death | Voriconazole | - |
| 5 | 62 | F | Lymphoma | 0,3 | 5,0 | <31,00 | NP | Negative | Lungs | Probable IA | Unevaluable | Voriconazole | - |
| 6 | 57 | F | AML | 0,1 | 0,3 | <31,00 | Positive | *Aspergillus fumigatus complex* | Lungs | Probable IA | Partial | Amphotericin B | - |
| 7 | 58 | M | AML | 0,8 | 0,9 | 33,13 | Positive | *Aspergillus fumigatus* | Lungs | Probable IA | Partial | Voriconazole | - |
| 8 | 45 | F | ALL | 0,1 | 3,9 | 177,25 | Negative | Negative | Lungs, Brain | Suspected | Unevaluable | Posaconazole | - |
| 9 | 57 | M | MDS | 3,9 | 1,7 | 135,18 | Negative | Negative | Lungs | Probable IA | Stable | Posaconazole | - |
| 10 | 66 | F | AML | 0,1 | 0,1 | <31,00 | NP | NP | Lungs | Proven IA | Partial | Voriconazole | - |
| 11 | 34 | F | AML | 0,6 | 0,9 | 38,59 | Negative | Negative | Lungs | Probable IA | Death | Amphotericin B | - |
| 12 | 43 | F | ALL | 0,1 | 0,5 | <31,00 | Positive | Negative | Lungs | Possible IA | Complete | Amphotericin B | - |
| 13 | 34 | M | ALL | 4,8 | NP | >500,00 | NP | NP | Lungs | Probable IA | Stable | Voriconazole | - |
| 14 | 66 | F | ALL | 2,2 | NP | 79,93 | NP | NP | Lungs | Probable IA | Unevaluable | Voriconazole | - |
| 15 | 46 | F | AML | 0,2 | 6,9 | 43,67 | Positive | Aspergillus fumigatus | Lungs | Probable IA | Partial | Voriconazole | - |
| 16 | 70 | M | AML | 0,1 | 0,3 | 305,59 | Negative | Negative | Lungs | Possible IA | Death | Amphotericin B | - |
| 17 | 62 | M | AML | 3,8 | 0,8 | 97,85 | Negative | Negative | Lungs, Abdominal | Probable IA | Partial | Voriconazole | - |
| 18 | 76 | F | AML | 0,1 | 0,1 | <31,00 | Positive | Negative | Lungs | Probable IA | Unevaluable | Voriconazole | - |
| 19 | 55 | M | CLL | 0,1 | 5,5 | <31,00 | Negative | Negative | Lungs | Probable IA | Unevaluable | Amphotericin B | - |
| 20 | 76 | M | ALL | 0,2 | 5,8 | <31,00 | Positive | Aspergillus niger | Lungs | Probable IA | Complete | Posaconazole | - |
| 21 | 62 | F | AML | 1,1 | 4,3 | 47,46 | Positive | Negative | Lungs | Probable IA | Partial | Posaconazole | **+** |
| 22 | 69 | F | AML | 0,2 | 1,9 | <31,00 | Negative | Aspergillus fumigatus | Lungs | Proven IA | Partial | Olorofim | - |
| 23 | 69 | M | MDS | 1,8 | 1,6 | 78,29 | Negative | Aspergillus fumigatus | Lungs | Probable IA | Partial | Isavuconazole | **+** |
| 24 | 29 | M | AML | 0,8 | 0,2 | <31,00 | NP | Negative | Lungs | Probable IA | Partial | Voriconazole  (+ SCY-078 vs placebo) | - |
| 25 | 64 | F | AML | 0,1 | 5,6 | 98,63 | Negative | Negative | Lungs | Possible IA | Death | Voriconazole | - |
| 26 | 68 | F | MDS | 0,1 | 3,1 | <31,00 | Positive | Negative | Lungs | Possible IA | Complete | Isavuconazole | - |
| 27 | 44 | F | CML | 0,1 | 4,1 | >500,00 | Negative | Negative | Lungs | Probable IA | Partial | Voriconazole | - |
| 28 | 76 | M | AML | 0,3 | 0,2 | <31,00 | Negative | Negative | Lungs | Possible IA | Partial | Posaconazole | - |
| 29 | 23 | F | Other | 1,6 | NP | 59,53 | NP | NP | Lungs | Probable IA | Complete | Isavuconazole | - |
| 30 | 58 | M | ALL | 3,6 | 6,1 | 223,07 | Negative | Negative | Lungs | Probable IA | Death | Voriconazole | - |
| 31 | 70 | M | MDS | 0,3 | 1,7 | <31,00 | Positive | Negative | Lungs | Probable IA | Complete | Amphotericin B | **+** |
| 32 | 69 | F | AML | 1,6 | NP | <31,00 | Positive | Negative | Lungs | Probable IA | Complete | Fosmanogepix | - |
| 33 | 76 | M | MDS | 0,0 | 0,2 | NP | Negative | Negative | Lungs | Possible IA | Partial | Voriconazole  (+ SCY-078 vs placebo) | - |
| 34 | 71 | M | AA | 3,3 | 4,8 | 160,82 | Positive | Negative | Lungs | Probable IA | Unevaluable | Voriconazole  (+ SCY-078 vs placebo) | - |
| 35 | 20 | M | ALL | 0,1 | 0,1 | 48,89 | Negative | Negative | Lungs | Possible IA | Partial | Amphotericin B | - |
| 36 | 55 | M | AML | 0,1 | 0,4 | <31,00 | Negative | Negative | Lungs | Possible IA | Partial | Posaconazole | - |
| 37 | 70 | M | MDS | 0,1 | 0,3 | NP | Negative | Negative | Lung | Possible IA | Unevaluable | Posaconazole | - |
| 38 | 66 | F | Lymphoma | 3,7 | 4,7 | 338,11 | Positive | Negative | Lungs | Probable IA | Stable | Isavuconazole | - |
| 39 | 51 | M | AML | 2,5 | 0,4 | 50,24 | Negative | Negative | Lungs | Probable IA | Partial | Isavuconazole | - |
| 40 | 31 | F | AML | 1,2 | 5,6 | NP | Negative | Negative | Lungs, Skin | Probable IA | Death | Isavuconazole | - |
| 41 | 55 | M | AML | 0,5 | 5,4 | 152,94 | Positive | Aspergillus fumigatus | Lungs | Probable IA | Unevaluable | Amphotericin B | - |
| 42 | 62 | F | CML | 0,1 | 4,3 | NP | Negative | Negative | Lungs | Probable IA | Stable | Voriconazole | - |
| 43 | 56 | F | AML | 1,2 | 6,3 | 474,96 | Positive | Aspergillus fumigatus | Lungs | Probable IA | Stable | Amfotericine B  + Caspofungin | - |
| 44 | 30 | M | AML | 0,6 | 0,1 | <31,00 | Negative | Negative | Lungs | Possible IA | Complete | Isavuconazole | - |
| 45 | 83 | M | AML | 0,1 | 1,8 | <31,00 | Negative | Negative | Lungs | Probable IA | Death | Amphotericin B | - |
| 46 | 71 | M | Lymphoma | 0,1 | 5,3 | NP | Negative | Negative | Lungs | Probable IA | Complete | Voriconazole | - |
