# Supplementary material for: Blood Mucorales PCR to track down Aspergillus and Mucorales co-infections in at-risk hematology patients: A case-control study
Source: Front Cell Infect Microbiol. 2022 Dec 8;12:1080921. doi: 10.3389/fcimb.2022.1080921 (PMC9774025; doi:10.3389/fcimb.2022.1080921)
Supplement: Supplementary Table 2 — Additional information regarding the patients (4 cases of invasive aspergillosis, 1 control patient) who had a positive Mucorales PCR in the diagnostic serum sample. BDG = beta-D-glucan; serumGM = galactomannan in serum; GalactoBAL = galactomannan in bronchoalveolar lavage fluid, AsperGenius = Aspergillus PCR (AsperGenius®, PathoNostics, Maastricht, The Netherlands); NP = not performed. [file Table_2.docx]

Supplementary table 2

*Additional information regarding the patients (4 cases of invasive aspergillosis, 1 control patient) who had a positive Mucorales PCR in the diagnostic serum sample.
BDG = beta-D-glucan; serumGM = galactomannan in serum; GalactoBAL = galactomannan in bronchoalveolar lavage fluid; AsperGenius = Aspergillus PCR (AsperGenius®, PathoNostics, Maastricht, The Netherlands); NP = not performed.*

| **Patient with + PCR** | **Mucorales PCR (cycles)** | **Interpretation of the result**  **(≤ 40,0 = positive)** | **SerumGM** | **BDG (pg/mL)** | **GalactoBAL** | **AsperGenius** | **CT chest** |
| --- | --- | --- | --- | --- | --- | --- | --- |
| A | 29,7 | Positive | 5,2 | >500,00 | 4,2 | NP | Nodules, >10 nodules, ground-glass opacities, pleural effusion |
| B | 33,8 | Positive | 1,1 | 47,46 | 4,3 | Positive | Nodular consolidations (3) with ground-glass surrounding zone, halo sign, pleural effusion |
| C | 36,7 | Positive | 1,8 | NP | 1,6 | NP | Nodules, >10 nodules |
| D | 32,5 | Positive | 0,3 | < 31,00 | 1,7 | Positive | Nodules, >10 nodules, nodular consolidation with ground-glass surrounding zone, halo sign |
| E | 29,4 | Positive | 0,1 | < 31,00 | 0,2 | Negative | Nodules, >10 nodules, nodular consolidation with ground-glass surrounding zone, halo sign, pleural effusion |
